# Medical–Legal and Psychosocial Considerations on Parental Alienation as a Form of Child Abuse: A Brief Review

**DOI:** 10.3390/healthcare10061134

**Published:** 2022-06-17

**Authors:** Oana-Maria Isailă, Sorin Hostiuc

**Affiliations:** 1 Department of Legal Medicine and Bioethics, Faculty of Dental Medicine, “Carol Davila” University of Medicine and Pharmacy, RO-020021 Bucharest, Romania; sorin.hostiuc@umfcd.ro; 2 “Mina Minovici” National Institute of Legal Medicine, RO-042122 Bucharest, Romania

**Keywords:** parental alienation, custody, child abuse

## Abstract

Parental alienation, an entity situated at the limit of psychiatry, sociology, and justice, still represents a controversial concept despite the legal dispositions that take it into account. The scope of this paper is to consider the relationship between parent and child, and child abuse from a psychosocial perspective, as well as to depict parental alienation, considered a form of child abuse, without omitting contradictory arguments which are also based on prudence in the minor’s interest, turning the attention to parental estrangement. Although parental alienation is not a psychiatric diagnosis per se and neither is parental estrangement, recognizing the difference between them is vital to adequately manage the situation at the time of establishing custody.

## 1. Introduction

The World Health Organization (WHO) refers to child abuse as “the abuse and neglect of children under 18 years of age, which includes all types of physical and/or emotional maltreatment, sexual abuse, deprivation, neglect, commercial exploitation or exploitation of any other nature which has the real or potential result of harm against the health, the survival, the development or the dignity of the child in the context of a relationship based on responsibility, trust or power.” [1].

According to the American Psychological Association, parental alienation represents “a child’s experience of being manipulated by one parent to turn against the other (targeted) parent and resist contact with him or her. This alignment with one parent and rejection of the other most often arises during child custody disputes following divorce or separation proceedings, particularly when the litigation is prolonged or involves significant antagonism between the parties.” [2]. Therefore it is considered parental alienation when the child rejects one of their parents and resists contact with them without a reasonable cause, expressing unjustified negative emotions [3,4].

Some authors hesitantly depict this phenomenon with respect to differentiating it from parental estrangement, referring to cases where a child has been subjected to real abuse by a parent they refused to interact with anymore, the refusal being justified [5].

The differences between parental alienation and parental estrangement criteria found in child abuse focus on contextual and legal issues that may have additional negative repercussions on the child’s psyche. An important role in this differentiation requires psychiatric medical–legal expertise.

From a medical standpoint, the phenomenon of parental alienation is not unanimously acknowledged, one of the arguments against it being its absence as a pathological entity per se from the international classifications: DSM, ICD, WHO, but it has been referred to indirectly [6].

From 2016, the phenomenon of parental alienation was legally identified as a form of child abuse on a national level [7]. The psychological abuse of a child is defined as a verbal or symbolic act of the parent or the caregiver which has, as a result, or a potential result, a significant psychological ailment. Physical and/or sexual abuse are not included in this category. Examples of psychological abuse against a child include the following: reprimanding; denigration; humiliation; threatening abandonment; hurting people or things that the child cares about; physically limiting the child; coercing the child to inflict pain on themselves; and excessive discipline, frequently or with increased duration, even if they do not represent physical abuse, through physical or non-physical means [8]. Also, according to DSM V, when it comes to the notion of “the child affected by the stress within the parental relationship”, it is recommended to use the term when it “focuses the clinical attention on the negative effects of discord in the parental relationship (for example increased levels of conflict, suffering or resentment) on the child within the family unit, including the effects on the mental or medical conditions of the child.” [8].

Psychiatry connects parental alienation to “pathological divorce”, the persons involved do not have psychiatric pathologies but the relationship becomes pathological, fuelled with hatred and disgust. Problems concerning custodial and visitation rights emerge in this context based on doubts regarding each person’s parental competence, in which case, according to the procedure, the opinion of a psychiatrist is required through a psychiatric legal medicine expertise report. The unjustified manifestations of a child refusing to interact with one of the parents, subjected to a pathological parental separation, raise the suspicion of parental alienation [6].

Parental alienation has repercussions on the child, as it annihilates their capacity to offer and receive affection from one of their parents. The child can develop: low self-esteem, lack of trust in oneself and others, depression, substance abuse, addiction, anxiety, self-sufficiency, uncertain attachment, feelings of loss, feelings of abandonment, feelings of guilt, and incapacity to respect authority, which are related to psychiatrical afflictions [9]. Also, negative effects were observed in the case of the alienated parent, including suicide attempts [10].

The scope of this paper is to depict the resorts and psychosocial consequences regarding the mental and emotional development of the child in the background of the parental alienation phenomenon.

## 2. Materials and Methods

We made an unsystematized review analyzing the literature on the topic of parental alienation with an emphasis on its consequences. We have also taken into consideration articles on the topic of child abuse from a psychological and social perspective. In this sense, we searched using Google Scholar, Doaj, and PubMed. The keywords used for the search were “parental alienation”, “child abuse”, and “child maltreatment”. The studies included in the review were the ones that brought to the fore the medical and psycho-social consequences of child abuse.

### 2.1. Child Abuse-Psychosocial Consequences

From a social and cultural standpoint, the relationship between parent and child has well-established elements related to the specific context. In terms of the parent–child relationship, some models are either unidirectional or bidirectional, depending on the influence of both. Unidirectional influences are found in all cultures during the first years of a child’s life, as the child is dependent on their parents, the age difference, and the availability of resources [11]. Overall, regardless of the social and cultural context, during childhood and adolescence, the relationship between parent and child is asymmetrical and is based on differences in resources to handle development requirements and needs. In time, this asymmetry can change because of the negotiation between child and parent concerning personal needs. From a Western standpoint, the purpose is to create the identity and autonomy of the child through certain changes which parents also go through, going from the role of “carer” to that of “facilitator of development”, where the bidirectionality of the relationship between parent and child prevails. In the development of the child’s identity within this relationship, emotional, attitudinal, conflictual, and functional aspects are taken into consideration [12].

In the case of abused children, attachment issues were found, as well as the tendency for them to ignore their emotions and cognitions [13] with an impact on adult life through the spectrum of inadequately responding to stressful situations in daily life [14]. Children subjected to adversity from an early age have a higher risk of developing psychiatric conditions such as depression and anxiety [15]. Developing in a dysfunctional, hostile, and disorganized environment was also associated with violence and impulsivity at an adult age [16].

Child abuse, regardless of context, is a determining factor in personality disorders, substance abuse, explosive intermittent disorder, and changes clinically manifested through aggression, such as domestic violence at an adult age [17].

### 2.2. Parental Alienation-Psychosocial Aspects

Parental alienation syndrome not only blocks the child’s capacity to give and offer affection to one of their parents, but it also brings fear and anger towards the alienated parent to the forefront [18].

Therefore, it is a form of psychological abuse, as the child is manipulated by the allied parent [19], and a form of contextual neglect of a child through deprivation of “normal parental quality” which ensures the child’s physical, emotional, developmental, and supervision needs are met by both parents [20]. In the case of parental alienation, manipulation of the child has the purpose to convince the child to resort to actions for which the child does not have interior resources, and it is done by depicting situations meant to instill fear and repulsion towards the alienated parent and/or placing the child in a situation meant to instill feelings of fear and sometimes even guilt to serve the emotional needs of the allied parent [21]. Psychological abuse in general, and this form of psychological abuse in particular, are difficult to objectify [19], requiring complex psychological, psychiatric, and medical–legal actions in this regard.

Child neglect can be physical and/or emotional. Physical neglect can occur through the absence of the basic needs of a child: food, clothing, physical safety, adequate supervision, and healthcare. Emotional neglect refers to not providing the child with basic emotional support. The absence of social needs or expecting a child to handle situations that go beyond the child’s capacity to understand as they are not yet mature are elements of parental neglect [21].

In the context of parental alienation, the child has to manage to sever the relationship with one of their parents based on beliefs that are assimilated from the exterior; this relationship would have contributed to ensuring the child’s basic physical and emotional needs [18,22].

**Figure 1:**
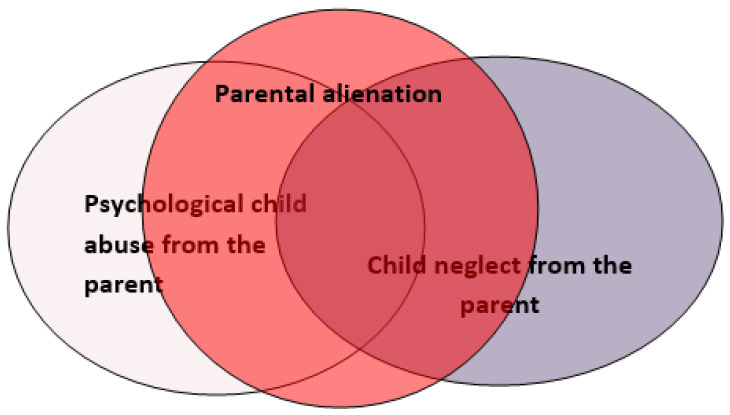
The relationship between parental alienation, psychological child abuse, and child neglect.

Regarding the abovementioned issues, the parental alienation phenomenon as a form of child abuse can be represented as follows (Figure 1).

These forms of abuse, in the given context, can lead to the child having feelings of uselessness, conditional worth, and lack of self-trust [3,23]. Lavadera et al., in the comparative study conducted on children who were parentally alienated in a sample with an average age of 11, has exclusively found in them the tendency to behave in a manipulating way, a lack of respect for authority, affective ambivalence, and a distorted perspective of the family dynamic [24]. Studies carried out in the case of adults who were parentally alienated during childhood have shown increased levels of anxiety [25], depression, low self-esteem, substance abuse, estrangement from their children, divorce, a feeling of lack of identity, and lack of belonging [3,26,27].

From a social standpoint, debates as to whether to create the diagnosis of parental alienation as a standalone psychiatric diagnosis have emphasized the risk of stigmatizing the child by labeling them with a mental disorder that is difficult to demonstrate and understand on a large scale. Furthermore, there are still controversial conceptual elements concerning the definition of the term and the diagnosis algorithms [28,29,30,31] considering the lesser-known facts and the subjective perception of parents (the parent who has custody of the child) and the child.

### 2.3. Parental Alienation versus Parental Estrangement

This terminology, although synonymous in the general perception, from a medical standpoint exposes different degrees: the justified versus the unjustified severing of the relationship between parent and child. The refusal to interact with one of the parents can occur in cases of divorce, refusal which is based on fear, or an episode of the revolt of the child in response to that context. The persistency of this refusal represents a serious issue that requires an investigation into the determining factors. The main possible reasons are parental alienation or parental estrangement. Parental estrangement is based on the reaction of a child as a consequence of concrete abuse from the targeted parent, whereas parental alienation does not have a justifiable underlying cause [32]. Although they are distinct phenomena with completely different approaches, parental alienation and parental estrangement, when looked at from an outside perspective, have the same result: the child’s absence of interaction with one of the parents who is divorced or in the process of divorce. Initially, the phenomenon of parental alienation was proposed as a “syndrome of parental alienation” so as to receive a code as a psychiatric diagnosis [18]. This aspect created disputes concerning the labeling of the child, and implicitly their stigmatization based on complex and difficult-to-objectify criteria, entrenched in studies with results which are difficult to generalize [33] but which would have an essential legal impact in obtaining custody of a child and implicitly in their development. According to Meier, the “syndrome of parental alienation” as a standalone diagnosis in the medical practice would “rather be used to negate real abuse than used to reduce psychological damage on minors” [34]. In this respect, presently, the term parental alienation is used when it comes to the unjustifiable refusal of a child to interact with the parent from whom they are separated as a result of divorce because of the manipulation of the alienating parent. The study conducted by Bernet et al. has underlined the fact that the child’s perception of the parent from whom they are alienated is more negative than in cases of parental estrangement, although in these cases there is a real reason to refuse interaction, the child, however, expresses ambivalent feelings. However, in cases of real and severe repeated abuse, the affective ambivalence towards the abusive parent was absent, a situation similar to cases of parental alienation and in which the degree of separation between the parent and the child is high [32]. Similar results were also presented by Clawar and Rivlin concerning the greater vehemence of alienated children in the rejection of the targeted parent [35].

Although parental alienation is not a psychiatric diagnosis per se, and neither is parental estrangement, separating them is vital for the child, in order to adequately approach the situation in the best interest of the child’s welfare during the moment of establishing custody. If distancing the child is based on a real history of abuse within the relationship between child and parent, the attitude of the child is justifiable [36]. The national legal framework imposes legal medicine expertise in such cases to avoid detecting false-positive cases of parental alienation [7,37] and to acknowledge the risk of maladaptation and the mental development of the child. Elements that can raise the degree of difficulty in the examination are the child being of a very young age, a long period of time having elapsed since the accusations were first made, and the presence of psychiatric pathologies in the parents/children [38].

### 2.4. Awareness and Support

Those involved in the phenomenon of parental alienation are the child, the alienating parent (the one with whom the child lives), and the alienated parent (the one from whom the child is separated). In addition, other people close to the child, generally relatives they trust and who contribute to the process of denigrating the alienated parent, also intervene in the process of parental alienation. The source of parental alienation is based on a complex range of extrinsic elements involved in the child’s manipulation to avoid interaction with the parent from whom they are separated [7,34].

In contrast to parental alienation, parental estrangement involves only the abused child and the abusive parent, with whom they refuse to interact. The cause of this child’s refusal is the intrinsic fear of parental abuse, amid aggression by the parent from whom they are separated, objectified or objectifiable. In order to avoid parental estrangement, it is necessary to stop the abuse of the child and to ensure adequate social, medical, and legal support [5].

In the therapeutic approach to parental alienation, traditional methods have not been shown to be effective [39]. According to Blotcky and Bernet, “The interventions must be aimed at (1) stopping the alienation by the offending parent, (2) clarifying and resolving the alienation in the child, and (3) repairing the child-rejected parent relationship. More traditional psychotherapies that do not target these specific areas are doomed to fail. The right therapy at the right time is critical” [40]. They also bring to the fore the so-called “reunification therapy” [39], which requires an experienced therapist in this field, and also the cessation of the alienating behavior of the parent, which may involve the separation of the child from the alienating parent [40].

On the other hand, according to Mercer, the methods based on the separation of the child from the preferred parent (ordered by the court) followed by specialized care and counseling, have not proved their effectiveness, being risky, and even harmful. The child needs both parents; the antagonistic decision to separate them from their alienating parent being radical and with potentially disastrous effects, given the possibility of weak parenting skills in the case of the alienated parent [41].

Given that parental alienation is a complex and insidious phenomenon, difficult to manage, with divergent, controversial opinions, it would be ideal to avoid it, bringing to the fore the child’s need to interact and benefit from the support of both parents, which requires adequate information at societal level in order to raise awareness of the phenomenon and its repercussions. Also, the first step towards adequate support is the correct forensic assessment of the family and the child.

## 3. Discussion

Parental alienation can be considered a complex form of child abuse in terms of its aspects involving psychological abuse and depriving the child of the support of one of the parents, which can be perceived as a form of neglect [21]. The consequences of the phenomenon at the social level can be the child’s social disinsertion, stigmatization, and family dysfunction in adulthood [26].

This article, which juxtaposes parental alienation and child abuse, emphasizes the importance of analyzing the parental situation when establishing custody, as well as later, in order to avoid instrumentalization and unjustified deprivation of the child from the available support of one of the parents. At the same time, it requires a rigorous analysis of the situation, so as not to confuse parental alienation with parental estrangement, in which case the child–parent interaction was or it still is abusive. Child abuse is found in both parental estrangement (but in an obvious form, frequently physical, committed by the parent from whom the child is separated) as well as in parental alienation (but in a more difficult form to observe, frequently psychological abuse, committed by the parent with whom the child lives) [32].

The results of this review have their applicability in the fields of professions that may intersect with the identification and/or management of the parental alienation phenomenon: physicians, psychologists, social workers, and lawyers.

## 4. Conclusions

The phenomenon of parental alienation is a complex, insidious form of child abuse situated on the limits of psychiatry, sociology, and justice. The role of medical–legal psychiatry is to catalyze the adequate management of this phenomenon, with the best interest of the child as the focus. The most important step towards preventing and combating parental alienation involves increasing the awareness of the phenomenon and providing adequate counseling for divorced parents and their relatives.

## Data Availability

Not applicable.
